# What makes staff consider leaving the health service in Malawi?

**DOI:** 10.1186/1478-4491-12-17

**Published:** 2014-03-19

**Authors:** Wanangwa Chimwaza, Effie Chipeta, Andrew Ngwira, Francis Kamwendo, Frank Taulo, Susan Bradley, Eilish McAuliffe

**Affiliations:** 1 University of Malawi, College of Medicine, Centre for Reproductive Health, Blantyre, Malawi; 2 Centre for Global Health, University of Dublin, Trinity College, Dublin, Ireland

## Abstract

**Background:**

Malawi faces a severe shortage of health workers, a factor that has contributed greatly to high maternal mortality in the country. Most clinical care is performed by mid-level providers (MLPs). While utilization of these cadres in providing health care is a solution to the current shortages, demotivating factors within the Malawian health system are pushing them into private, non-governmental, and other non-health related positions. This study aims to highlight these demotivating factors by exploring the critical aspects that influence MLPs’ intention to leave their jobs.

**Methods:**

This descriptive qualitative study formed part of the larger Health Systems Strengthening for Equity (HSSE) study. Data presented in this paper were collected in Malawi using the Critical Incident Analysis tool. Participants were asked to narrate an incident that had happened during the past three months which had made them seriously consider leaving their job. Data were subjected to thematic analysis using NVivo 8 software.

**Results:**

Of the 84 respondents who participated in a Critical Incident Analysis interview, 58 respondents (69%) indicated they had experienced a demotivating incident in the previous three months that had made them seriously consider leaving their job. The most commonly cited critical factors were being treated unfairly or with disrespect, lack of recognition of their efforts, delays and inconsistencies in salary payments, lack of transparent processes and criteria for upgrading or promotion, and death of patients.

**Conclusion:**

Staff motivation and an enabling environment are crucial factors for retaining MLPs in the Malawian health system. This study revealed key ‘tipping points’ that drive staff to seriously consider leaving their jobs. Many of the factors underlying these critical incidents can be addressed by improved management practices and the introduction of fair and transparent policies. Managers need to be trained and equipped with effective managerial skills and staff should have access to equal opportunities for upgrading and promotion. There is need for continuous effort to mobilize the resources needed to fill gaps in basic equipment, supplies, and medicine, as these are critical in creating an enabling environment for MLPs.

## Background

Malawi is one of the countries facing severe staffing shortages in the health sector and, as such, has a very low capacity to meet a minimal level of health care. The most recent health worker to patient densities (2009) are 0.019 physicians and 0.343 nursing and midwifery personnel per 1,000 population [1], and there are ongoing vacancies across all nursing and clinical cadres [2]. In addition to these shortages, the health sector in Malawi is also faced with uneven distribution of staff, with more than half of the doctors working in the four central hospitals and a few in district hospitals [3]. Even in district hospitals that have a doctor, administrative and leadership tasks take up a considerable portion of the doctor’s time, resulting in the necessity for much of the clinical work to be completed by mid-level cadres [4].

Mid-level providers (MLPs) are health workers with at least two to three years of post-secondary school health-care training, who undertake tasks usually carried out by doctors and nurses, such as clinical or diagnostic functions [5]. Clinical officers complete three-year direct entry training plus a one-year internship, and are found in district and central hospitals; medical assistant training is two years plus a one-year internship, after which they can upgrade to clinical officers, and they are typically found in health centers [6]. Registered nurse-midwives perform the tasks commonly undertaken by doctors; and nurse-midwife technicians and enrolled nurses perform tasks commonly undertaken by registered nurses or nurse-midwives. While utilization of MLPs in providing health care is a solution to the current shortage of health workers and a way of increasing skilled health care to the population, their numbers are less than adequate. The 2010 needs assessment of emergency obstetric and newborn care services in Malawi [7] found unacceptably high vacancy rates for MLPs. Only 28% of targeted clinical officers and 40% of targeted enrolled nurse midwife/nurse midwife technician cadres were found, yet these cadres carry out the bulk of emergency obstetric care (EmOC). In addition, some of the internationally recognized cadres, such as nurses, have over the years migrated from Malawi to work in other countries, such as the United Kingdom, in search of better prospects [8].

Among its considerable attempts to address the human resources crisis Malawi developed a six-year Emergency Human Resources Programme (EHRP) which was launched in 2004 as one pillar of the Programme of Work, implemented through the Health Sector Wide Approach (SWAp) [9]. This aimed to improve staff recruitment and retention through a donor-supported 52% salary top-up for key cadres, increased pre-service training, plus a range of incentives for all health workers. These additional incentives took the form of rural allowances, housing, and transport, based on the premise that increased remuneration and benefits retain people in their jobs [10,11]. By 2009 the health worker to population density had risen from the 2004 figure of 0.87 per 1,000 to 1.44 per 1,000, representing a 66% increase as a result of the EHRP [2]. However, these new levels still fell below the African region average (1.91 per 1,000) and the World Health Organization’s critical threshold (2.5 per 1,000) for delivering essential health services [12], showing the depth of Malawi’s human resources crisis.

Increasing health worker migration into private, urban, tertiary facilities is undermining provision of appropriate rural, primary care [13]. Common factors contributing to internal migration of health workers include low pay, lack of resources, poor working conditions, inadequate supervision, and lack of career progression [10]. However, many of these serve as general demotivating factors that are commonly present and are known to the health workers even before they choose to train as health professionals. This study undertook a more in-depth exploration of the critical juncture at which a health worker decides to leave his/her job, in order to expose those factors over and above the demotivators that have been documented as being present in health facilities in Malawi [14], that is, to understand what happens to cause a health worker to decide that they can no longer continue in their job. To date there has been little research on the types of specific incidents that push health workers to the brink and cause them to seriously contemplate leaving their posts. This study explored such incidents among mid-level cadres working in EmOC settings in Malawi.

## Methodology

### Study design and sample

This qualitative exploratory study was part of the larger Health Systems Strengthening for Equity (HSSE) study. The HSSE study aimed to expand the evidence base on effective use of mid-level health workers in EmOC. Data for this element of the study were collected through anonymous Critical Incident Analysis interviews. Critical incidents are incidents or events that are critical to the person’s view of a particular phenomenon or problem. This is a technique that is commonly used for collecting incidents that the respondent feels have been critical to his or her experience of a job [15]. Once the incident has been recorded the interviewer uses probing questions to elicit the details of the incident and the respondent’s reactions and feelings about the incident. Incidents that caused the health worker to consider leaving the job can be explored in this way in order to identify factors that are critical to health workers’ decisions on whether to leave or remain in a particular employment.

Data for the broader HSSE study were collected in 25 of Malawi’s 28 districts. Eighty-eight health facilities providing EmOC were visited. All types of facility were included: government district hospitals; Christian Health Association of Malawi (CHAM) health facilities; and health centers providing EmOC. To avoid overburdening health workers, three districts (10 facilities) that had recently taken part in another Human Resources for Health study were excluded.

An information circular was sent to facility managers detailing the project and they were contacted one week to 10 days later for their decision on participation. When a positive response was received the research team visited the facility. In each facility teams first introduced themselves and the study to the facility and/or maternity in-charge. They inquired about the number of staff currently working in the maternity unit, as well as any staff who might have temporarily been assigned to another unit (for example, the outpatient department or the reproductive child health unit). The maternity and/or facility in-charges also helped to identify the clinical staff (that is, doctors, clinical officers, and medical assistants) who are called for emergency procedures, such as cesarean sections. Teams recorded the number of staff in each cadre to ensure that there would be adequate representation of each cadre in the total sample.

All relevant health workers in the selected facilities were given a Participant Information Leaflet detailing the nature of the project and their possible involvement during the data collection process. A member of the research team was always on site to answer any questions about the project during the two days allocated for each site.

The eligibility criteria for the broader HSSE study required all participants to have performed at least one of the EmOC signal functions^a^ in the three months prior to the study, so prospective participants indicated which ones they had performed using a list shown to them by the data collectors. A total of 631 health workers took part in the main data collection. A purposive sample was used to identify a subset of these health workers for the Critical Incident study. To be eligible for this element they had to respond positively when asked if they had experienced a specific incident at work that caused them to become demotivated or even to think about leaving their job, and be prepared to describe this incident in an interview. When the research team was satisfied that staff fully understood the nature of the project and what was required of them, and that they were both willing and eligible to continue, staff were asked to provide written consent.

### Data collection

Data collection took place from October to December 2008. The research team included three experienced qualitative interviewers and two clinical officers who had all been trained in the study and its methodology prior to data collection. They were asked to interview one participant in each facility and to try to target all cadres providing EmOC. To ensure good participation and response, the interviewers had to be sensitive to the prospective interviewee’s work schedule, be flexible with regards to interview time, and use a private, quiet place for the interview. A total of 84 interviews were carried out, all of which happened to be with MLPs.

The interviews were recorded and transcribed by the interviewers. Most were in English, but a few were in Chichewa (the most common local language spoken in Malawi) because the interviewees felt more comfortable conversing in Chichewa. The Chichewa interviews were transcribed in Chichewa by the interviewers and later translated into English by researchers proficient in both languages. The transcripts were later imported into an NVivo 8 software database for thematic analysis.

### Data analysis

Data analysis was carried out by three researchers from the College of Medicine in Malawi (WC, EC, and AN) and a qualitative researcher in Ireland (SB). A coding template, based on the literature and discussions about the data, was developed. All four researchers spent one week together in Malawi to perform the initial coding face-to-face, discuss emerging themes, and validate their coding. Further analysis was carried out by the team in Malawi, with additional input from SB. The coding framework was revised as new nodes emerged. These were clustered into relevant top-level themes, then a process of synthesis was used to draw out key findings and themes.

### Ethical approval

The study was approved by the Institutional Review Boards of Columbia University, New York and the College of Medicine, Malawi; and by the Global Health Ethics Committee, Trinity College, Dublin.

## Results

Eighty-four MLPs narrated critical incidents that had caused them to become demotivated. However, this element of the research was interested only in those who had seriously contemplated leaving their job as a result of the incident they narrated. Fifty-eight (69%) participants met this criterion and their interviews are the focus of this paper. Of these, thirty- five were employed in government owned facilities and twenty-three were working in CHAM establishments. The majority were based in hospitals, with only six respondents based in health centers. Over 70% (n = 42) were female and 50% (n = 29) were nurse-midwife technicians (NMT). The remaining respondents were seven clinical officers (CO), seven medical assistants (MA), eight registered nurses and registered nurse-midwives (RN/M), and seven enrolled nurse-midwives (EN/M).

The factors that caused these MLPs to think seriously about leaving their jobs can be divided into two broad categories: (1) factors that cause staff to feel under-valued; and (2) factors that lead to poor quality patient care.

## Staff feeling under-valued

### Management issues

The most common reason given for intention to leave was poor management. Respondents reported that they had bad relationships with managers who in most cases ill-treated them. In one incident a medical assistant had her belongings thrown out of her house because she had gone for a holiday when she felt she was due one but her in-charge did not want her to go.

*‘So I stayed like 2 years without getting my annual leave, so when the new doctor came I told him my problem and he said I should wait until we have more staff. And then we received more staff after the MCHS* [Malawi College of Health Sciences] *graduated some students, we received about three clinicians so we were about eight in total, and I asked for my annual leave and he refused to sign for the forms. So when I had my off duty I went home and there I found out that my sister had an accident there she was unable to walk, the leg was swollen and was in pain. So I decided to call my boss and I called him, said “This is the situation at home, can I still have some few more days leave?” He said “Yes, okay”…that’s when they thought of throwing my things out of my house when I was not coming back from holiday, but then I told them I am coming this weekend so they said, “Oh, you are getting late, you are one day late, we will throw your things out”*. (MA, 2022)

Cases of managers shouting at their juniors were frequently reported. Staff in management positions were also reported to be stubborn and to not want their juniors to air their views. In general, MLPs felt that they were not adequately supported, not treated equitably, and their efforts were not recognized by their managers. This caused these health workers to be demotivated and to contemplate leaving.

### Financial issues

Finances were frequently mentioned during the interviews. One respondent was demoralized because his name did not appear on the list of people to receive locum allowances.

*‘It was the week when we were receiving our allowances in terms of locums and alike, it was unfortunate that I didn’t appear on the locum list… I was very depressed, I was very demotivated, because after working hard within that month I did not receive any locum, not even any appreciation from the management’*. (EN, 2172)

In another incident a clinical officer reported that at one time he wanted to quit because the administration at the facility where he worked took a long time to raise his salary after the government had sanctioned salary increments with immediate effect. A similar incident was also reported by a nurse-midwife who indicated that he wanted to leave because his salary was not raised for a long time after he got promoted. In addition, two medical assistants complained of late receipt of their salaries.

*‘…just imagine the salaries were due by the 5th of October, they did nothing until the 20th when I had confronted them. If I had not, nothing would have happened, so I feel they have some bad motives towards their workers. Because I don’t know why they should hold somebody’s money! So I was seriously demotivated… if they had done nothing I was quitting’*. (CO, 3091)

A nurse-midwife technician described being demoralized and wanting to quit his job because he felt there was some injustice in the way salaries were paid in CHAM health facilities, in that there were differences in the amounts staff received despite having the same qualification. In one incident a nurse-midwife technician reported her intention to leave her job because she was very bitter when she discovered that other working colleagues were getting more top-up allowances than her. Further, an enrolled nurse-midwife reported that she had wanted to leave her job at one time because she was paid less locum allowance than she was supposed to receive after working for many hours.

### Housing and infrastructure

There were several respondents who narrated incidents of poor housing that caused them to be demoralized and intend to leave their jobs. In one incident a nurse was frustrated after being told by the District Nursing Officer (DNO) that she would be accommodated in a motel because there was no house available for her. Concerning the same issue of housing, two other nurse-midwives complained that they felt demotivated because they were staying in a house that had not been maintained for a long time.

*‘…basically there is one major problem currently… that is concerning accommodation. As you can see right away I am residing in a house… which has completely dilapidated doors and rusty corrugated iron sheets. You know this is now rainy season. It is leaking…’* (MA, 3172)

### Promotion and upgrading

Respondents narrated incidents in which they had considered leaving their jobs because they were frustrated by the lack of recognition or promotion they received after upgrading their qualifications to a higher cadre. Responses from interviewees clearly indicated that there was no system in place that enabled staff to be automatically upgraded or promoted even after undergoing upgrading training. A nursing sister in one of the facilities indicated that one reason why service providers could not be promoted automatically after going for upgrading training was because the hospital management was not authorized to upgrade staff. Instead, management waited for authorization from the Health Service Commission, which could take a long time. Respondents reported that in some instances service providers were given extra responsibilities and duties, but these were not reflected in promotion to the new grade.

*‘I gave them the notification of results. Up to now I haven’t heard from them…to be given the salary that I deserve just because I have more responsibilities now than when I was a nurse technician’.* (NMT, 2132)

In another incident a nurse-midwife wanted to leave to work in a non-governmental organization (NGO). She had moved from a CHAM facility to work in a government facility, but was told that she would be put at a lower grade than when she was at CHAM, despite her qualifications.

*‘…there was this issue, I wanted to be recognized since I wanted to be promoted. I went to school for my upgrading and after that upgrading I thought maybe my grade will be a bit raised, but unfortunately nothing has happened so far despite all our initiatives. But no promotion has been given. So I was really demotivated, I just wanted to leave at a certain time’.* (NO, 3072)

Respondents also reported lack of transparency from managers when there were opportunities for staff to apply for upgrading. Four nurse-midwife technicians, a medical assistant, and a clinical officer all reported they were not notified about promotion interviews and as a result were not able to apply. Other respondents considered leaving their jobs because there were no opportunities for upgrading in the facilities where they were working. A medical assistant working in a CHAM facility indicated he wanted to leave his job because he had come to the realization that the chances of upgrading in a CHAM institution were slim. In one incident a clinical officer was very disappointed because he had undergone a two-year training programme and was promised that after finishing he would go to Mzuzu University to specialize in surgery. However, this did not happen, which had caused him to think of leaving his job to go to an NGO.

*‘…and after hearing the news that we are not going to Mzuzu we were demoralized and we reached a point of wanting to resign and going to maybe Medicins san Frontiers and just concentrate on things like ARVs and HIV issues, because we have been demoralized a lot’.* (CO, 5112)

### In-service training

While respondents indicated that opportunities for in-service training were available it was frequently reported that there was unfairness in the choice of who should attend training in many facilities. Two nurse-midwife technicians, a registered nurse, and a medical assistant indicated that there was favoritism in that often it was the same people who were chosen to go for in-service training.

*‘…our sister in-charge, she makes her own decisions. Hmmm! Like when they are some in-service trainings, she chooses the one she wants, not considering that the one she is choosing already attended other in-service trainings, so I was frustrated’.* (NMT, 3061)

## Factors that lead to poor quality patient care

### Lack of resources

A general lack of drugs, equipment, and other supplies affected the performance of clinical duties. The majority of staff indicated that sometimes they found that essential drugs were out of stock and there was nothing that they could do since they had no alternative.

*‘I have been here for a year and seven months, but* [since] *my arrival day we have been talking of medications, medications. Now I am fed up, I just feel that I have to quit. Because if you tell your patient you have no medication it is as if you don’t know what you are doing. I can’t work without medication and we only treat simple conditions’.* (MA, 2191)

Some of the common equipment that was in short supply in both labor and general wards included macintosh (a rubberised waterproof sheet used to cover hospital beds to prevent liquids soaking the mattress), curtains for privacy, blood pressure (BP) machines, glucometers, suction machines, HIV testing kits, and delivery packs. It was reported that the lack of these items severely affected the quality of MLPs’ work.

*‘Sometimes you may want to take BP for the patient in the labor ward and yet you don’t have BP machines. Maybe they have been locked in the seniors’ offices and you call them that I want the BP machine and they say “at this time I cannot come as I’m far away”. It really makes your performance low’.* (CO, 1081)

### Staff shortages and workload

Overwhelming lack of staff in maternity and too great a workload were major factors in causing respondents to seriously contemplate leaving their jobs. Many staff commented on the difficulties of not having anyone else to help them, or of having to run around looking for senior staff to assist them when they were alone on the wards. There was a clear intersection with fear of being involved in a maternal death.

Staff reported being expected to attend to a number of wards at the same time. One nurse-midwife technician (NMT, 1031) described being left alone in the clinic to attend to the antenatal clinic, outpatient department (OPD) and the labor ward. She had prioritized a patient in the labor ward because ‘*…this one in labor needed me to assist her… I had to be with this one definitely…*’ but the patients in the OPD started shouting at her, which demoralized her to the extent that she wanted to leave her job. In another incident a nurse-midwife technician reported feeling burnt out due to too great a workload, which had resulted from receiving extra patients with gynecological complications who had been sent from a nearby district hospital that was under reconstruction.

Being left to cope alone, particularly at night, was a serious concern for a number of respondents. Two nurses described incidents where they were refused assistance when things got out of hand because of a policy of only assigning one nurse per night shift. Another nurse-midwife technician reported that she had thought of transferring to a different facility because one night she was left alone to attend to the labor and postnatal wards. Seeing that the labor ward was full she had asked for extra help from the matron, who told her there was no one to help her out because of staff shortages. In a very similar incident another respondent described a situation of having responsibility for eight patients in the labor ward, three of whom were complicated cases. When she had asked for help the matron had told her that only one nurse is allocated on night duty.

*‘…there was a time when I was in maternity ward then I had about eight patients but I was alone. So I call the doctor because one patient had fetal distress… the other one developed PPH* [postpartum hemorrhage] *and I had a referral from another health center with a retained placenta. When I called the other people to help, like calling the DNO* [District Nursing Officer] *telling her what was happening and in the situation…she told me on night duty we allocate one… So that day in the morning I said “why can’t I leave because if somebody dies in my hands I will feel guilty the rest of my life”. So maybe I should leave’.* (NMT, 3143)

### Negative interactions with other staff or colleagues

Issues to do with negative interactions with fellow working colleagues were frequently cited as causing respondents to contemplate leaving their jobs. In one incident a nurse wanted to leave because she felt mistreated by a clinician who ignored her patient as an emergency when the patient was bleeding severely from retained placenta. When the nurse attempted to persuade the clinical officer to intervene he called the nurse stupid and told her that even other clinicians in the hospital complained about her behavior.

A clinical officer recounted an incident where a fellow clinician blamed him for the death of a girl whose guardian had deliberately hidden information from him that her daughter was suffering from abortion complications. This had made him want to leave his job. In another incident a nurse wanted to leave a health facility because a fellow workmate had given his name to the community after a maternal death occurred in his hands and the husband of the deceased kept threatening him.

### Death of patient

Several respondents indicated that the preventable death of a patient had left them so demotivated that they had intended to leave their job. These deaths occurred either because of mismanagement of a case, due to action or in-action of a co-worker, or because of lack of resources. In one such incident a nurse-midwife was demoralized because a child had died when a laboratory assistant who was supposed to do the necessary tests did not come to do his duties. A similar incident was also reported by another nurse-midwife whereby a maternal death occurred because of mismanagement by a clinician who was supposed to perform a cesarean section on the patient but did not do it. In another incident a woman with postpartum hemorrhage bled to death because an ambulance did not come in time for referral.

Respondents also narrated death-related incidents that made them want to leave their jobs because the management, fellow staff or the Nurse’s Council, blamed them for the deaths of patients. These incidents were reported by a clinical officer, two enrolled nurse-midwives, and two nurse-midwife technicians. These respondents indicated that the deaths were usually because of delays by the patient in getting to the health facility, or that staff had done all they could to save the patients but they had failed.

## Discussion

Participants in this study described a range of demotivating factors associated with health worker retention, many of which are consistent with previous literature in this area. These factors directly affect MLPs’ motivation or create a disabling environment, making it difficult for these cadres to perform their duties well. However, using the Critical Incident methodology revealed key ‘tipping points’ that push health workers to the brink of leaving their job. These are critical incidents over and above the standard demotivators that are known quantities in this context.

Inadequate management support ranked highly as the most commonly mentioned push factor for intention to leave. Critical incidents occurred when MLPs felt that they were treated unfairly or with disrespect, or when their efforts were not recognized by their managers. This echoes the results of previous studies with these cadres in Malawi [16] and is a cause of considerable demotivation for MLPs. This finding reveals inadequacies in the management skills of the type of staff put in managerial positions in health facilities. In low-income countries management positions in the health sector are often occupied by untrained managers, particularly at lower levels [17]. This study’s findings suggest the importance and need to equip managers in the health sector with good managerial skills. There is evidence that skilled managers have the ability to motivate their employees and are responsible for lobbying on behalf of health workers [18]. In a review on leadership styles and outcome patterns for the nursing workforce and work environments, it was revealed that leaders who relate well with their staff are able to understand their issues and work concerns, and to support and invest in them and their abilities. This leads to completion of the tasks required to achieve the common goal; in the case of health care this is the provision of excellence in patient care [19]. In addition, managers with good skills have the ability to retain staff. ‘Lead’ managers, who use caring habits such as encouraging, respecting and trusting staff, can provide better staff support and more satisfying work conditions; they may manage to retain staff for longer than ‘boss’ managers who tend to use more negative habits such as blaming, criticizing and punishing [20].

Respondents in this study felt the need for more effective human resource management. This finding is in line with a 2010 evaluation of the EHRP [2]. Management and leadership development is one of the strategies stipulated in the current Malawi Health Sector Strategic Plan to harness human resource capacity development efforts [21]. Efforts have been made by the Ministry of Health to train health workers in management. The College of Medicine’s (COM) Master of Public Health programme provides a mechanism for the Ministry of Health whereby district and central hospital health management team members are trained [22]. The COM and Kamuzu College of Nursing both offer Bachelor of Science in Health Management courses. Despite these efforts there are still gaps in management training since these courses have limited numbers. There is therefore a need to introduce more management courses in other health worker training institutions as well.

Several studies have shown that low or poor salary is a major factor causing intention to leave among health workers [14,23,24]. The in-depth nature of this study has revealed that it is not the level of salary *per se* that is the causative factor; rather the critical push comes when staff are not given salary that is due to them. This includes late salary payments, delays in increasing salaries after a promotion, or inconsistencies in locum allowance payments. This is an additional injustice and a demotivator for MLPs considering that their salaries are already low. Therefore ensuring that salary payments are consistent and timely is one way of retaining health workers in the health system.

Lack of opportunities for upgrading and promotion were a key concern in this study, consistent with previous research on motivation and retention of health workers. For example, securing future career development and desire for self-development were some of the factors that motivated community health workers in Bangladesh not to leave their jobs [25]. A review on motivation of health workers in developing countries found education and training opportunities to have strong motivating effects in fifteen of the papers that were reviewed [18]. It was indicated that training enables workers to take on more demanding duties and to achieve personal goals of professional advancement, as well as allowing them to cope better with the requirements of their job. This was found to be especially important for young health professionals. The critical factors in the present study, however, concerned the lack of systems to automatically promote staff who had completed upgrading training or to match salaries to qualifications or grade. Additionally, the lack of transparent processes or clear criteria in the provision of opportunities for upgrading or promotion and the perceived unfairness of decisions on who accessed these opportunities were a key tipping point for some staff.

Staff shortage is the factor which leads to huge workload and, coupled with lack of resources, results in poor quality of care performed by MLPs. Staff joining the Malawian health system are aware of the human resources and material constraints in the system and deal with these on a daily basis. The critical juncture at which MLPs in this study felt they could no longer continue was frequently related to their inability to work to professional standards, or fear of being involved in, or blamed for, the preventable death of a patient. Deaths of some patients in such an environment are inevitable and this eventually leads to poor staff motivation and job dissatisfaction, which can finally make a health worker decide to leave, thus creating a vicious cycle of staff attrition. A study conducted on managing the work environment of MLPs found that inadequate resources lead to job dissatisfaction, dissatisfaction with one’s profession, thinking about leaving one’s job and, more worryingly, to active plans to seek other employment [26]. Similar findings have also emerged in several other studies on health worker attrition and retention [14,23,24]. These findings indicate the importance of a good enabling environment in the retention of MLPs. Emphasis and effort should continuously be put on lobbying for more funding to improve the working conditions for health workers, to ensure adequate supplies of equipment and medicines, and to create a more enabling environment.

This study was conducted in 2008 when the Malawi government was in the fifth year of implementing its EHRP, which aimed to improve staffing levels in the health sector. Thus some of the issues raised had, in theory, already been tackled in the EHRP, for example issues of incentives, staff shortages and training or upgrading. However other issues, such as poor working environment, lack of resources, and poor management at service level, were not addressed. It is clear from this study that many of these demotivating factors still exist, causing health workers to consider leaving their jobs. Exit interviews with health workers could be used to identify the specific factors that finally push health workers to quit and to inform the development of targeted interventions to address these. Participants in this study were asked to articulate the factors that ultimately resulted in a decision not to leave their post. A considerable body of data was generated and will be presented in a forthcoming paper.

Evaluation of the EHRP indicates a success story in terms of increasing the health worker density [2], but attention needs to be paid to valuing and supporting existing health workers if the achievements of the EHRP are to be maintained. Furthermore, losing MLPs to other sectors has economic implications. For instance, replacing an employee who has left for another job can be costly in terms of recruitment and training costs. Beyond the financial cost there is also the loss of knowledge and commitment associated with long-term employees. The shortage of the key cadres delivering EmOC, such as clinical officers and enrolled nurse midwife/nurse midwife technician cadres, also poses a serious challenge for the government of Malawi in reaching Millennium Development Goal 5. It has significant implications for the quality of care that can be delivered, in that the number of facilities equipped to offer EmOC 24 hours a day is reduced, contributing to higher maternal mortality rates [27].

## Conclusion

Staff motivation and an enabling environment are crucial factors for retaining MLPs in the Malawian health system. This study revealed key ‘tipping points’ that drive staff to seriously consider leaving their jobs. Many of the factors underlying these critical incidents can be addressed by improved management practices and the introduction of fair and transparent policies. Managers need to be trained and equipped with effective managerial skills and staff should have access to equal opportunities for upgrading and promotion. There is need for continuous effort to mobilize the resources needed to fill gaps in basic equipment, supplies, and medicine, as these are critical in creating an enabling environment for MLPs.

### Limitations

Data for this study were drawn from a subset of MLPs who were willing to discuss an incident that had caused them sufficient distress to make them seriously consider leaving their post. While it is evident that the issues they recounted were important to them it is unclear whether these concerns are representative of the larger MLP population. Seventy percent of participants were female and half were nurse-midwife technicians, with a further 12% who were enrolled nurse-midwives. It may well be the case that there are differences in the saliency of different factors across cadres and grades, or between genders, and this would be an interesting area to explore further. Furthermore, only 10% of participants were from health centers, where the pressures could be expected to differ to those seen in hospitals. A possible explanation is that staff in a small facility may have been inhibited from participating in the research as their engagement could have impacted negatively on patient care. Additionally, there may have been reluctance to be involved in an interview if their supervisors or colleagues knew it was about an incident that caused them to consider leaving their job. In an effort to be sensitive to this the interviewers explained that the focus of the Critical Incident interview was to find out about the critical things that cause most unhappiness in the job, by exploring a specific incident that caused the participant to become demotivated or even to think about leaving. In a small health center, with fewer health workers, staff may have felt too exposed to take part. However, there is sufficient agreement across the narratives collected and coherence with previous work done by the authors in Malawi to suggest that these issues are relevant to staff retention and are likely to apply to these cadres more widely.

An additional limitation was the technical complexity of carrying out the data analysis in Malawi and Ireland. Every effort was made to ensure inter-rater reliability, including the use of the initial face-to-face coding workshop, agreeing node descriptions, and use of frequent conference calls and communication to maintain the integrity of the analysis.

## Endnote

^a^Basic EmOC is comprised of seven signal functions: 1. Administer parenteral antibiotics; 2. Administer uterotonic drugs; 3. Administer parenteral anticonvulsants for pre-eclampsia and eclampsia; 4. Manual removal of placenta; 5. Removal of retained products of conception; 6. Assisted vaginal delivery; 7. Neonatal resuscitation. An additional two signal functions indicate comprehensive EmOC: 8. Perform surgery (for example, cesarean section); 9. Perform blood transfusion.

## Competing interests

The authors declare they have no competing interests.

## Authors’ contributions

WC participated in the data collection/analysis and drafted this paper. EC participated in the study data analysis and contributed to the paper. AN participated in the data collection/analysis and contributed to the paper. FT participated in the study design, data collection, and data analysis. FK participated in the study design, data collection, and data analysis. SB participated in the study design, data analysis, and contributed to the paper. EM participated in the study design, data analysis, and contributed to the paper. All authors read and approved the final manuscript.

## References

[B1] Global Health Observatory Data Repository [http://apps.who.int/gho/data/node.main.A1444?lang=en] 10.1080/02763869.2019.1693231 32069199

[B2] O'Neil M Jarrah Z Nkosi L Collins D Perry C Jackson J Kuchande H Mlambala A Evaluation of Malawi’S Emergency Human Resources Programme 2010 Cambridge, MA: Department for International Development (DFID), Management Sciences for Health (MSH), and Management Solutions Consulting (MSC) 1 87

[B3] Observatory MHW Human Resources for Health Country Profile Malawi 2010 Lilongwe: Government of Malawi

[B4] van Amelsfoort JJC van Leeuwen PAM Jiskoot P Ratsma YEC Surgery in Malawi - the training of clinical officers Tropical Doctor 2010 40 74 76 10.1258/td.2009.090068 20305097

[B5] World Health Organization, Global Health Workforce Alliance Mid-level health providers: a promising resource to achieve the health Millennium Development Goals 2010 Geneva: World Health Organization

[B6] Lobis S Mbaruku G Kamwendo F McAuliffe E Austin J de Pinho H Expected to deliver: alignment of regulation, training, and actual performance of emergency obstetric care providers in Malawi and Tanzania Int J Gynaecol Obstet 2011 115 322 327 10.1016/j.ijgo.2011.09.008 22036057

[B7] Ministry of Health, UNICEF, UNFPA, World Health Organization, AMDD Malawi 2010 EmONC Needs Assessment Final Report 2010 Lilongwe: Ministry of Health

[B8] Record R Mohiddin A An economic perspective on Malawi’s medical ‘brain drain’ Global Health 2006 2 12 10.1186/1744-8603-2-12 17176457 PMC1769362

[B9] Ministry of Health Malawi Human Resources in the Health Sector: Towards a Solution 2004 Blantyre: Government of Malawi

[B10] Palmer D Tackling Malawi’s Human Resources Crisis Reprod Health Matters 2006 14 27 39 10.1016/S0968-8080(06)27244-6 16713877

[B11] Kawonga M Fonn S Achieving effective cervical screening coverage in South Africa through human resources and health systems development Reprod Health Matters 2008 16 32 40 10.1016/S0968-8080(08)32403-3 19027620

[B12] World Health Organization Working Together for Health: the World Health Report 2006 2006 Geneva: World Health Organization

[B13] McAuliffe E Manafa O Maseko F Bowie C White E Understanding job satisfaction amongst mid-level cadres in Malawi: the contribution of organisational justice Reprod Health Matters 2009 17 80 90 10.1016/S0968-8080(09)33443-6 19523585

[B14] Manafa O McAuliffe E Maseko F Bowie C MacLachlan M Normand C Retention of health workers in Malawi: perspectives of health workers and district management Hum Resour Health 2009 7 65 10.1186/1478-4491-7-65 19638222 PMC2722569

[B15] MacLachlan M McAuliffe E Critical incidents for psychology students in a refugee camp: implications for counselling Couns Psychol Q 1993 6 3 10 10.1080/09515079308254487

[B16] Bradley S McAuliffe E Mid-level providers in emergency obstetric and newborn health care: factors affecting their performance and retention within the Malawian health system Hum Resour Health 2009 7 14 10.1186/1478-4491-7-14 19228409 PMC2657772

[B17] Wiskow C The Effects of Reforms on the Health Workforce 2006 Geneva: World Health Organization

[B18] Willis-Shattuck M Bidwell P Thomas S Wyness L Blaauw D Ditlopo P Motivation and retention of health workers in developing countries: a systematic review BMC Health Serv Res 2008 8 247 10.1186/1472-6963-8-247 19055827 PMC2612662

[B19] Cummings GG MacGregor T Davey M Lee H Wong CA Lo E Muise M Stafford E Leadership styles and outcome patterns for the nursing workforce and work environment: A systematic review Int J Nurs Stud 2010 47 363 385 19781702 10.1016/j.ijnurstu.2009.08.006

[B20] Stagnitti K Schoo A Dunbar J Reid C An exploration of issues of management and intention to stay: allied health professionals in South West Victoria, Australia J Allied Health 2006 35 226 232 17243438

[B21] Ministry of Health Malawi Malawi Health Sector Strategic Plan 2011-2016 2011 Lilongwe: Ministry of Health

[B22] Ministry of Health Malawi Health SWAp Donor Group GTZ Human Resources/Capacity Development Within the Health Sector - Needs Assessment Study 2007 Lilongwe: Ministry of Health

[B23] Ndetei DM Khasakhala L Omolo JO Incentives for Health Worker Retention in Kenya: an Assessment of Current Practice 2008 Harare: EQUINET

[B24] Awofeso N Improving health workforce recruitment and retention in rural and remote regions of Nigeria Rural Remote Health 2010 10 1319 20136347

[B25] Rahman SM Ali NA Jennings L Seraji MHR Mannan I Shah RZ Mahmud AB Bari S Hossain D Das MK Baqui AH Arifeen SE Winch PJ Factors affecting recruitment and retention of community health workers in a newborn care intervention in Bangladesh Hum Resour Health 2010 8 12 10.1186/1478-4491-8-12 20438642 PMC2875202

[B26] McAuliffe E Bowie C Manafa O Maseko F MacLachlan M Hevey D Normand C Chirwa ML Measuring and managing the work environment of the mid-level provider–the neglected human resource Hum Resour Health 2009 7 13 10.1186/1478-4491-7-13 19228391 PMC2655277

[B27] Gerein N Green A Pearson S The implications of shortages of health professionals for maternal health in sub-Saharan Africa Reprod Health Matters 2006 14 40 50 10.1016/S0968-8080(06)27225-2 16713878

